# The *Plasmodium falciparum* RING Finger Protein *Pf*RNF1 Forms an Interaction Network with Regulators of Sexual Development

**DOI:** 10.3390/ijms26125470

**Published:** 2025-06-07

**Authors:** Afia Farrukh, Sherihan Musa, Ute Distler, Stefan Tenzer, Gabriele Pradel, Che Julius Ngwa

**Affiliations:** 1 Division of Cellular and Applied Infection Biology, RWTH Aachen University, 52074 Aachen, Germany; afia.farrukh@bio2.rwth-aachen.de (A.F.); musa@bio2.rwth-aachen.de (S.M.); ngwa.che@bio2.rwth-aachen.de (C.J.N.); 2 Institute of Immunology, University Medical Centre of the Johannes-Gutenberg University, 55131 Mainz, Germany; ute.distler@uni-mainz.de (U.D.); tenzer@uni-mainz.de (S.T.)

**Keywords:** malaria, *Plasmodium falciparum*, gametocyte, sexual development, zinc finger protein, RBUL, transcription, interactome

## Abstract

RNA-binding E3 ubiquitin ligases (RBULs) provide a link between RNA metabolic processes and the ubiquitin proteasome system (UPS). In humans, RBULs are involved in various biological processes, such as cell proliferation and differentiation, as well as sexual development. To date, little is known about their role in the protozoan parasite *Plasmodium falciparum*, the causative agent of malaria tropica. We previously identified a novel *P. falciparum* RBUL, the RING finger E3 ligase *Pf*RNF1, which is highly expressed during gametocyte development. Here, we conducted BioID-based proximity interaction studies to unveil the *Pf*RNF1 interactome. We show that in immature gametocytes, *Pf*RNF1 forms an interaction network that is mainly composed of RNA-binding proteins, including the translational repressors DOZI and CITH and members of the CCR4-NOT complex, as well as UPS-related proteins. In particular, *Pf*RNF1 interacts with recently identified regulators of sexual development like the zinc finger protein *Pf*MD3, with which it shares the majority of interactors. The common interactome of *Pf*RNF1 and *Pf*MD3 comprises several uncharacterized proteins predominantly expressed in male or female gametocytes. Our results demonstrate that *Pf*RNF1 engages with RNA-binding proteins crucial for sex determination in gametocytes, thereby linking posttranscriptional regulation with the UPS.

## 1. Introduction

Malaria, caused by *Plasmodium* parasites, leads to 597,000 deaths annually, with *Plasmodium falciparum* being responsible for the most lethal infections [1]. Malaria pathogenesis is linked to the proliferating blood stages of the parasite, while the sexual stages, particularly the gametocytes, play a critical role in disease transmission by *Anopheles* mosquitoes. The sexual phase of *P. falciparum* starts with sexual commitment, which occurs in a small fraction of asexual blood-stage parasites and is triggered by environmental factors such as nutrient depletion. This process is particularly regulated by the Apetala 2 (Ap2) transcription factor AP2-G and leads to gametocytogenesis. During asexual blood-stage replication, the AP2-G-encoding gene is silenced by heterochromatin protein 1 (HP1) but becomes activated following the removal of HP1 by gametocyte development protein 1 (GDV1). AP2-G then enables sexual commitment and the development of gametocytes through the cascading activation of gametocyte-specific genes (e.g., reviewed in [2,3,4]).

AP2-G reprograms asexual blood-stage parasites for sexual development, but how it drives male and female gametocyte differentiation is unclear. *Plasmodium* lacks sex chromosomes, suggesting that epigenetic and epitranscriptomic factors control sex determination. Several AP2 proteins were shown to regulate sex identity in gametocytes, including AP2-FG and AP2-O3, which promote female gene profiles while repressing male genes, and AP2-G5, which modulates male development [5,6,7,8]. Furthermore, RNA-binding proteins (RBPs) play a critical role during gametocyte development by regulating posttranscriptional processes. Some plasmodial RBPs, such as DOZI (development of zygote inhibited), CITH (worm CAR-I and fly Trailer Hitch), and PUF2 (Pumilio and Fem-3 binding factor 2), repress transcripts in female gametocytes and store them for later use following parasite transmission to mosquitoes, when the zygote needs to develop in the mosquito midgut (e.g., [9,10,11,12]; reviewed in [13,14]).

A still under-investigated group of RBPs with roles in gametocytogenesis are zinc finger proteins (ZFPs) (reviewed in [15]). In general, ZFPs, specifically those bearing C3H1 motifs, play critical roles in RNA metabolic processes like mRNA splicing, polyadenylation, export, and translation, as well as ubiquitination and transcriptional repression (e.g., reviewed in [16,17]). Recently, we characterized two C3H1-ZFPs with important functions during the development of male *P. falciparum* gametocytes, *Pf*MD3 (male development protein 3) and *Pf*ZNF4 (zinc finger protein 4). Both ZFPs were originally identified by us during a transcriptomic screen for genes deregulated upon treatment of gametocytes with the histone deacetylase inhibitor Trichostatin A (TSA) [18]. While parasites deficient in *Pf*MD3 are impaired in male gametocyte maturation, *Pf*ZNF4 deficiency blocks male gametogenesis through the downregulation of male-enriched genes, particularly those associated with axoneme formation [19,20]. The plasmodial MD3 is part of a group of sexual regulators that were identified during a global screen of barcoded *P. berghei* mutants and predicted to be important for the development of male and female gametocytes [21].

Another ZFP identified during the screening for TSA-deregulated genes of *P. falciparum* gametocytes is the RING finger domain-containing RNA-binding E3 ubiquitin ligase (RBUL) *Pf*RNF1 [18]. Generally, RBULs are key players in linking the RNA metabolism with the ubiquitin–proteasome system (UPS), and in humans, they are involved in various biological processes, such as cell proliferation and differentiation, as well as sexual development (reviewed in [22,23]). Here, we identified the *Pf*RNF1 interactome during the gametocytogenesis of *P. falciparum* using BioID-based interaction studies and demonstrated a comprehensive interaction network of *Pf*RNF1 with gametocyte-specific RBPs and sexual development regulators.

## 2. Results

**Figure 1:**
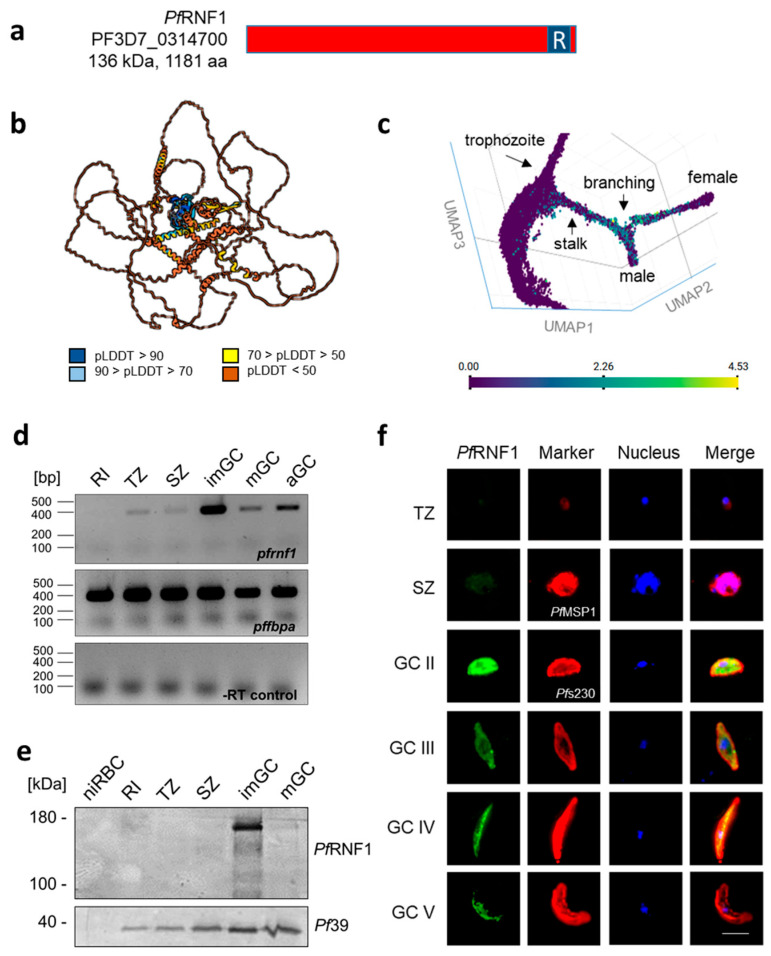
*Pf*RNF1 is expressed during gametocyte development in *P. falciparum*. (**a**) Schematic depicting *Pf*RNF1. The RING finger domain (R) is highlighted. (**b**) Predicted 3-D structure of *Pf*RNF1. The 3-D structure was generated using the AlphaFold database. (**c**) Single-cell gene expression of *Pf*RNF1 across the stalk and branching phases of gametocyte development. The image depicts a UMAP plot obtained from the Malaria Cell Atlas database with the color code representing gene expression levels (log_2_ counts). (**d**) Transcript expression of *Pf*RNF1 in blood-stage parasites. Complementary DNA from rings (RI), trophozoites (TZ), schizonts (SZ), and immature (imGC), mature (mGC), and 30 min post-activation (aGC) gametocytes of WT NF54 was subjected to diagnostic RT-PCR using *pfrnf1*-specific primers. The transcript amplification of aldolase-encoding *pffbpa* was used as housekeeping control, and samples without reverse transcriptase (-RT) served as genomic DNA controls. (**e**) Protein expression of *Pf*RNF1 in blood-stage parasites. Lysates from the RI, TZ, SZ, imGC, and mGC stages of WT NF54 were immunoblotted with mouse anti-*Pf*RNF1.2 antisera to detect *Pf*RNF1 (~136 kDa). Non-infected red blood cells (niRBCs) served as a negative control, and immunoblotting with rabbit antisera directed against the endoplasmic reticulum-resident *Pf*39 (~39 kDa) served as loading control. (**f**) Localization of *Pf*RNF1 in gametocytes. Methanol-fixed TZ, SZ, and GC II–V stages of WT NF54 were immunolabeled with mouse anti-*Pf*RNF1.2 antisera (green). Asexual blood stages and gametocytes are highlighted with rabbit antisera directed against *Pf*MSP1 and *Pf*s230, respectively (red); nuclei are highlighted with Hoechst 33342 nuclear stain (blue). Bar, 5 µm.

*Pf*RNF1 is a 136-kDa protein with a C-terminal RING zinc finger domain (Figure 1a). AlphaFold protein structure analysis predicted a globular protein with several central helices and a conserved C-terminal ring finger domain (Figure 1b). Analysis of single-cell transcriptomics using publicly available data from the Malaria Cell Atlas revealed low numbers of *pfrnf1*-expressing cells in the sexual commitment and stalk phase of gametocyte development and increasing numbers of *pfrnf1*-positive cells during branching and in gametocytes of male and female identity (Figure 1c). Semi-quantitative RT-PCR using RNA from rings, trophozoites, and schizonts, as well as from immature, mature, and activated gametocytes, showed high *pfrnf1* transcript levels in gametocytes compared to the those in asexual blood stages, with particularly high levels in immature gametocytes (Figure 1d and Figure S1a).

The stage specificity and subcellular localization of *Pf*RNF1 were investigated using existing anti-*Pf*RNF1.1 and anti-*Pf*RNF1.2 antibodies (Figure S1b). Western blotting of lysates generated from rings, trophozoites, and schizonts as well as from immature and mature gametocytes highlighted prominent *Pf*RNF1 levels in immature gametocytes, while only weak bands were detected in the other parasite stages (Figure 1e). Indirect immunofluorescence assays (IFAs) localized *Pf*RNF1 to the cytoplasm and nucleus of the developing and activated gametocytes and confirmed peak *Pf*RNF1 levels in immature stage II gametocytes (Figure 1f and Figure S2). The expression data were in accord with our previous reports on *Pf*RNF1 [18].

To determine the *Pf*RNF1 interaction network, we generated a transgenic line episomally expressing a *Pf*RNF1-GFP-BirA fusion protein. Blood-stage parasites were transfected with the vector pARL-*Pf*RNF1-*pffnp*a-GFP-BirA [20,24], whereby the expression of *Pf*RNF1-GFP-BirA was controlled by the gametocyte-specific *pffnpa* promotor (Figure S3a). Diagnostic PCR confirmed the presence of the respective vector in the transgenic line (Figure S3b). IFA using anti-GFP antibody demonstrated the presence of GFP-tagged *Pf*RNF1 in the maturing gametocytes (Figure S4a). Western blot analysis confirmed the presence of the *Pf*RNF1-GFP-BirA fusion protein with an expected molecular weight of ~200 kDa in gametocyte lysates of line *Pf*RNF1-*pffnpa*-GFP-BirA (Figure S4b). Protein biotinylation in the immature gametocytes was verified by Western blotting, following incubation of the transgenic parasites with 50 µM biotin for 24 h. Immunoblotting with alkaline phosphatase-conjugated streptavidin resulted in multiple bands of potential biotinylated proteins, including a band running at ~200 kDa, likely representing biotinylated *Pf*RNF1-GFP-BirA (Figure S4c). No prominent bands were detected in lysates of biotin-treated WT NF54 parasites.

Immature gametocytes of line *Pf*RNF1-*pffnpa*-GFP-BirA were treated with 50 µM biotin for 24 h and subjected to mass spectrometry-based proximity-dependent biotin identification (BioID-MS) to identify the *Pf*RNF1 interactome. BioID-MS resulted in the identification of 233 significantly enriched hits in immature *Pf*RNF1-*pffnpa*-GFP-BirA gametocytes (Tables S1 and S2). For further analyses, we excluded proteins with predicted signal peptides, which are likely to be secreted, resulting in a total of 226 putative interactors. These included components of the ribosomal subunits and the UPS, proteins involved in translation initiation (eIFs) and repression (e.g., CITH, DOZI), as well as in mRNA decay (e.g., CCR4-NOT components).

**Figure 2:**
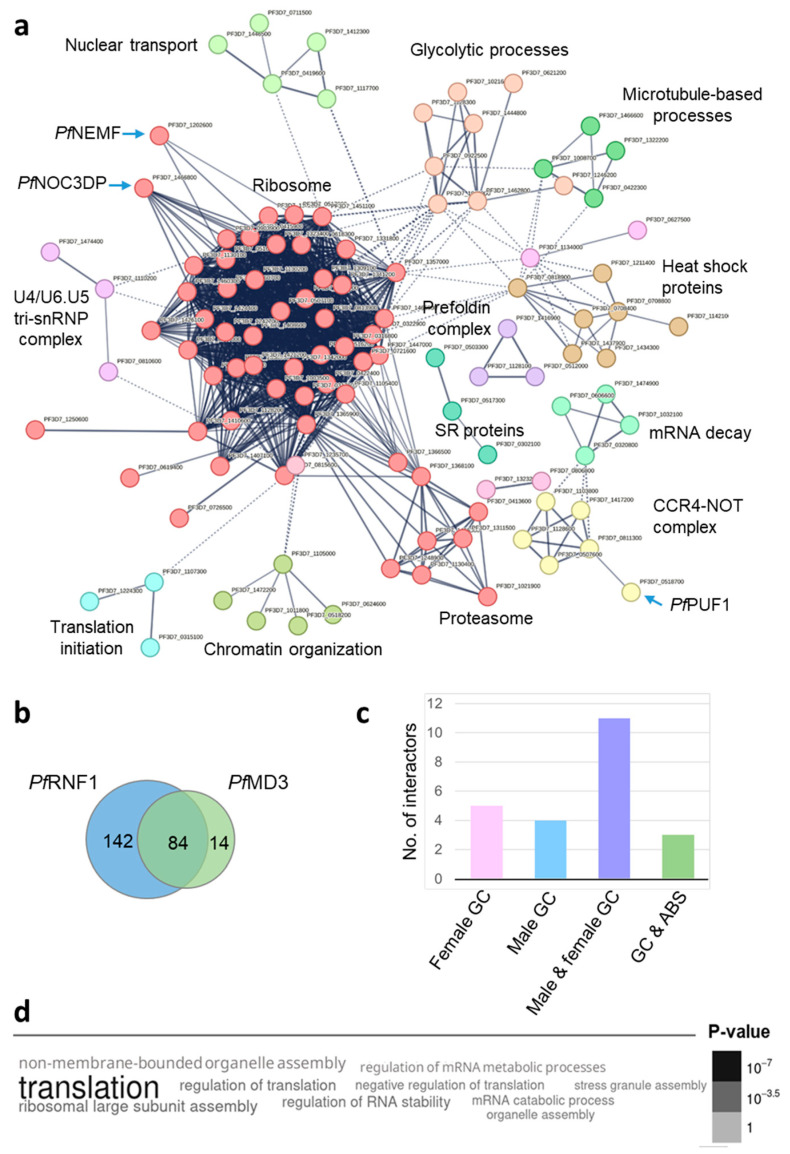
*Pf*RNF1 forms an interaction network composed of RBPs and translational regulators. (**a**) Network analysis of the *Pf*RNF1 interactors in immature gametocytes. A protein–protein network of the 226 putative *Pf*RNF1 interactors in immature gametocytes was generated using the STRING database and the Markov Clustering algorithm. Disconnected nodes were excluded. Selected clusters and interactors are highlighted (for a high-resolution image, see Table S3). (**b**) Venn diagram depicting interactors shared between *Pf*RNF1 (226 interactors) and *Pf*MD3 (98 interactors) [18]. (**c**) Bar diagram depicting numbers and sex specificity of interactors shared between *Pf*RNF1 and *Pf*MD3 with high expression in gametocytes. The expression profiles were visually analyzed using the Malaria Cell Atlas database. (**d**) Word cloud depicting the biological processes of interactors shared between *Pf*RNF1 and *Pf*MD3. GO enrichment analysis (*p*-value cutoff = 0.001) was performed using the PlasmoDB database.

The putative *Pf*RNF1 interactors were subjected to STRING-based analyses to investigate protein–protein interaction networks using the Markov Clustering algorithm. A total of 15 clusters were identified, 8 of which included ≥ 4 proteins (Figure 2a; Table S3). The most prominent cluster comprised ribosomal proteins and involved a satellite cluster of proteasomal components (red cluster). Two proteins stood out from this cluster, showing multiple interactions with other cluster members, i.e., the NOC3 (nucleolar complex-associated protein 3) domain-containing protein PF3D7_1466800 (henceforth termed *Pf*NOC3DP) and the nuclear export mediator factor *Pf*NEMF (PF3D7_1202600). Further clusters included proteins with roles in chromatin organization (olive cluster), nuclear transport (green cluster), and mRNA decay (light green cluster); two smaller clusters contained proteins related to splicing, i.e., three SR proteins (medium sea green cluster) and three spliceosomal U4/U6.U5 tri-snRNP components (orchid cluster). These clusters together indicated a strong link between *Pf*RNF1 and RNA metabolic processes. A CCR4-NOT complex cluster (yellow cluster) linked to the RBP *Pf*PUF1 and a cluster of three translation initiation components (cyan cluster) together with clusters of heat shock proteins (dark goldenrod cluster), and prefoldin subunits (medium purple cluster) additionally connected *Pf*RNF1 with processes of proteostasis. Other clusters of the interactome network included components of glycolytic processes (brown cluster) and V-type ATPases (hot pink cluster).

During a recent study on the C3H1-ZFP *Pf*MD3, we identified *Pf*RNF1 as its interactor by BioID analysis and validated the protein–protein interaction via co-immunoprecipitation assays [20]; here, *Pf*MD3 was conversely identified as an interactor of *Pf*RNF1. We therefore compared the interactomes of *Pf*RNF1 (226 proteins) and *Pf*MD3 (98 proteins) and identified 84 interactors shared by both ZFPs (Figure 2b; Table S2). To investigate the potential sex specificity of the interactors shared by *Pf*RNF1 and *Pf*MD3, we visually analyzed their transcriptomic profiles, which are publicly available at the Malaria Cell Atlas database. Of the 84 interactors, transcripts of 11 proteins were particularly abundant in gametocytes of both sexes; 5 were highly abundant in female and 4 in male gametocytes (Figure 2c). Three interactors had high transcript levels in gametocytes but higher transcript levels in the asexual blood stages; 61 interactors had comparable transcript levels in asexual blood stages and gametocytes or were solely expressed in the asexual blood stages. Notably, the shared interactors found in gametocytes included the majority of the recently identified group of regulators of male and female gametocyte development, i.e., GD1, FD1, FD2, FD4, and MD2 [21]. Gene ontology (GO) enrichment analyses assigned the shared interactors in particular to the biological processes of translation and regulation of RNA stability (Figure 2d).

**Figure 3:**
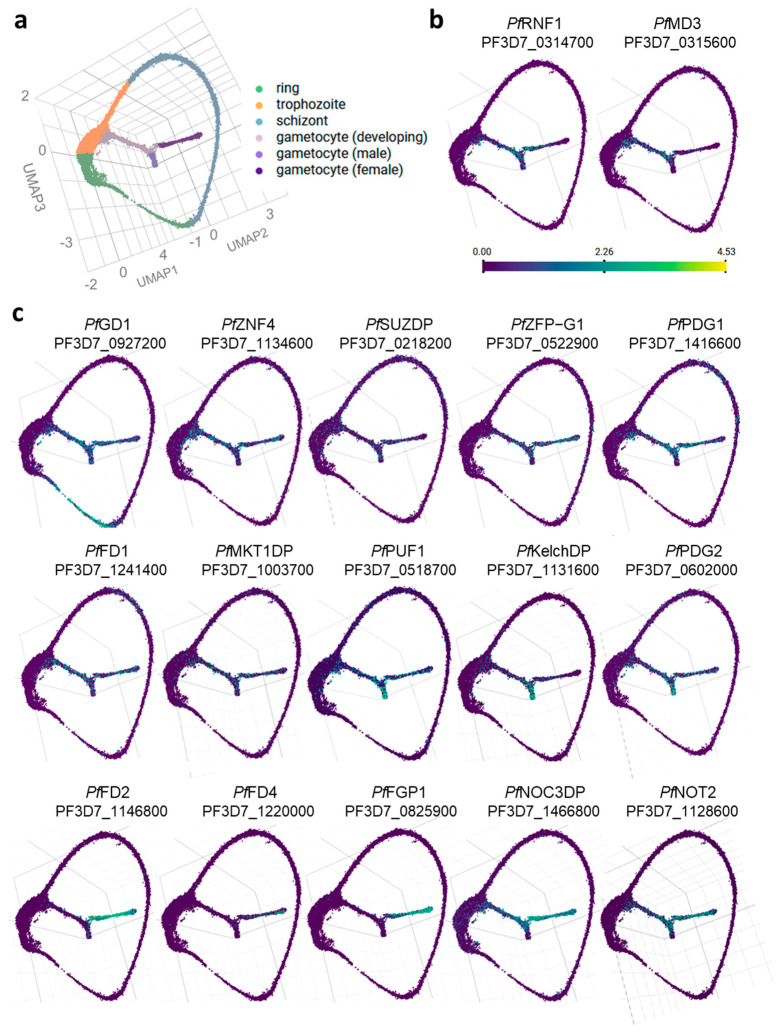
Single-cell transcriptome profiling of interactors shared between *Pf*RNF1 and *Pf*MD3 reveals novel gametocyte-specific proteins. (**a**) Representative image of single-cell transcriptomes across the asexual blood stages, developing gametocytes, and sexually differentiated male and female gametocytes. (**b**) Single-cell gene expression of *Pf*RNF1 and *Pf*MD3. (**c**) Single-cell transcriptome profiling of 15 interactors shared by *Pf*RNF1 and *Pf*MD3 with high expression in gametocytes. The images depict UMAP plots generated using the Malaria Cell Atlas database; the color code represents the respective gene expression levels (log_2_ counts).

Transcriptomic profiling was performed in more detail for *Pf*RNF1, *Pf*MD3, and 15 selected interactors using the Malaria Cell Atlas database (Figure 3a). *Pf*RNF1 and *Pf*MD3 transcript expression was depicted in gametocytes during development independently of sex (Figure 3b). Interactors of the two bait proteins that were particularly expressed in female gametocytes according to visual interpretation from the respective Malaria Cell Atlas UMAP plot included, in addition to *Pf*FD2, *Pf*FD4, and the CCR4-NOT component *Pf*NOT2, two unknown proteins, namely, PF3D7_0825900 (henceforth termed female gametocyte protein *Pf*FGP1) and the above-mentioned *Pf*NOC3DP (Figure 3c; Table S2). The four proteins that were transcriptionally highly expressed in males included the RBP *Pf*PUF1, the structural inner membrane complex (IMC)-associated protein *Pf*PIP1, the Kelch domain-containing protein PF3D7_1131600 (henceforth termed *Pf*KelchDP), and a yet unknown protein, PF3D7_0602000 (henceforth termed protein of developing gametocytes *Pf*PDG2). Notably, *Pf*PDG2 was previously described as a C3H1-ZFP [15,25]; however, a distinct zinc finger domain could not be annotated. The 11 proteins, which were highly expressed in both male and female gametocytes, included *Pf*RNF1, *Pf*ZNF4, *Pf*FD1, *Pf*MD3, *Pf*MDV1 (male development gene 1), and the structural IMC proteins *Pf*PIP2 and *Pf*PIP3. Further proteins in male and female gametocytes were the MKT1 domain-containing protein PF3D7_1003700 (henceforth termed *Pf*MKT1DP), the C3H1-ZFP PF3D7_0522900 (henceforth termed *Pf*ZFP-G1), the SUZ domain-containing protein PF3D7_0218200 (henceforth termed *Pf*SUZDP), and a yet unknown *Plasmodium* protein, PF3D7_1416600 (henceforth termed protein of developing gametocytes *Pf*PDG1). Three proteins exhibited high transcript expression in gametocytes as well as in asexual blood-stage parasites, namely, *Pf*GD1, *Pf*a35-2, and ornithine aminotransferase *Pf*OAT (Figure 3c, Table S2). Notably, *Pf*MD2 appeared to be expressed in two transcript variants, both of which showed low expression levels and were thus not included in the evaluation.

## 3. Discussion

Our combined data show that *Pf*RNF1 is an RBUL of gametocytes that forms a comprehensive interaction network with other ZFPs and recently identified regulators of gametocyte development. The expression of *Pf*RNF1 starts in the stalk phase of gametocyte development and continues during branching and the early sex identity phase with peak levels at stage II gametocyte stages. As previously shown by us, the *pfrnf1*-encoding gene associates with acetylated H3K9, and both *Pf*RNF1-specific transcript and protein levels increase following treatment of gametocytes with TSA [18], suggesting that its expression during sexual development is epigenetically regulated.

The most prominent interactors of *Pf*RNF1 can be divided in two groups. The first group comprises components of the UPS, like proteasome subunits, the ubiquitin-like protein PF3D7_0922100, and the ubiquitin-specific protease PF3D7_0904600. The second group comprises various types of RBPs. These include the ALBA family members ALBA1, ALBA3, and ALBA4, which have functions in mRNA homeostasis and translational regulation [26,27,28], as well as components of the CCR4-NOT core complex like CAF1, CAF40, NOT1-G, NOT1, and NOT2. The CCR4-NOT complex is a conserved large, multifunctional assembly of proteins that function in mRNA decay [29]. The plasmodial components of the CCR4-NOT complex have mainly been studied in *P. yoelii*. It was demonstrated by loss-of-function studies that *Py*CCR4-1, *Py*NOT1-G, and *Pf*CAF1 play crucial roles during gametocyte development and gametogenesis by regulating mRNAs important for these processes [30,31]. Other RBPs that interact with *Pf*RNF1 are associated with translational repression, such as CITH, DOZI, and PABP1. These RBPs store mRNAs that encode proteins required for the development of the mosquito midgut stages in cytosolic granules, and the transcripts are only introduced to protein synthesis at the onset of gametogenesis (reviewed in [14]). A further interacting RBP is *Pf*PUF1. Notably, its deficiency leads to a sharp decline in late-stage gametocytes and a sex-ratio shift towards males [32]. Considering the fact that single-cell transcriptomics assign *Pf*PUF1 particularly to the male branch, a function of *Pf*PUF1 in repressing male transcripts can be considered.

*Pf*RNF1 also interacts with the C3H1-ZFP *Pf*MD3 [20], a regulator of male development. We recently showed that a lack of *Pf*MD3 significantly impairs gametocyte maturation and leads to a sex-ratio shift towards females [20]. We now demonstrate that both ZFPs, *Pf*RNF1 and *Pf*MD3, share the majority of interactors. The majority of the shared interactors have predicted functions in translation and RNA stability and include in particular members of gametocyte development regulators, as originally identified in *P. berghei*, i.e., GD1, FD1, FD2, FD4, and MD2 [21] as well as *Pf*ZNF4, a C3H1-ZFP crucial for male gametogenesis [19]. To be highlighted is the putative interaction of *Pf*RNF1 and *Pf*MD3 with *Pf*GD1, a regulator of female gametocyte development. *P. berghei* parasites lacking the orthologous *Pb*GD1 show a sex-ratio shift towards males, comparable to the above-mentioned loss-of-function phenotype of *Pf*PUF1. Co-immunoprecipitation assays using *Pb*GD1 as bait revealed several interactors that were also shared between *Pf*RNF1 and *Pf*MD3, such as the RBPs CITH, PUF1, NOT-1G, PABP1, PDG2, and MKT1DB; the sex development regulators FD1, FD2, and FD4; and the ATP-dependent RNA helicase DBP1 and 14-3-3I (PF3D7_0818200) [21]. It is worth mentioning that in yeast, MKT1 is a posttranscriptional regulator that interacts with the poly(A)-binding protein Pab1 to regulate the translation of the mating-type switching endonuclease HO [33], suggesting a comparable role of MKT1DB during the sex determination of *P. falciparum* gametocytes. *Pf*RNF1 was also identified in a protein interaction network with the male development regulator *Pf*MD1, which is also an interactor of *Pf*MD3. *Pf*MD1 is a component of cytoplasmic granules and involved in male gametocyte development, with the N-terminus of the regulator being crucial for a male fate, while the LOTUS domain at the C-terminus guides male gametocytogenesis [34]. The combined data pinpoint *Pf*RNF1 as part of a network composed of RBPs and translational regulators involved in sex determination, most likely by promoting and repressing or degrading transcripts important for male or female fate. In accord with these findings, a recent single-cell transcriptomics analysis reported that the *Pf*RNF1-encoding gene is targeted by AP2-G5, a transcription factor regulating male development [8].

In conclusion, we provide evidence that *Pf*RNF1 is a multifunctional RBUL that links the UPS with RBPs to control the posttranscriptional machinery of *P. falciparum* gametocytes. We hypothesize that *Pf*RNF1 is part of a regulatory network that balances the threshold traits of gene products required for sex identity during the branching phase of gametocyte development. In humans, RBULs are currently investigated as novel targets for anticancer therapy (reviewed in [35]), which gives rise to hope that plasmodial RBULs could represent target structures for antimalarials and transmission-blocking agents in future studies.

## 4. Materials and Methods

### 4.1. Gene Identifiers

The following PlasmoDB gene IDs were assigned to the genes and proteins examined in this study: *Pf*RNF1 (PF3D7_0314700); *Pf*39 (PF3D7_1108600); *Pf*s230 (PF3D7_0209000); *Pf*AMA1 (PF3D7_1133400); *Pf*CCp2 (PF3D7_1455800); *Pf*FBPA (PF3D7_1444800); *Pf*FD1 (PF3D7_1241400); *Pf*FD2 (PF3D7_1146800); *Pf*FD4 (PF3D7_1220000); *Pf*FGP1 (PF3D7_0825900); *Pf*FNPA (PF3D7_1451600); *Pf*GD1 (PF3D7_0927200); *Pf*KelchDP (PF3D7_1131600); *Pf*MD3 (PF3D7_0315600); *Pf*MSP1 (PF3D7_0930300); *Pf*MKT1DP (PF3D7_1003700); *Pf*NOC3DP (PF3D7_1466800); *Pf*NOT2 (PF3D7_1128600); *Pf*PDG1 (PF3D7_1416600); *Pf*PDG2 (PF3D7_0602000); *Pf*PUF1 (PF3D7_0518700); *Pf*SUZDP (PF3D7_0218200); *Pf*ZFP-G1 (PF3D7_0522900); *Pf*ZNF4 (PF3D7_1134600).

### 4.2. Antibodies

The following primary antibodies were used in the study: mouse anti-GFP (Roche, Basel, Switzerland); rabbit anti-*Pf*s230 (BioGenes, Berlin, Germany); rabbit anti-*Pf*39 (Davids Biotechnology, Regensburg, Germany); rabbit anti-*Pf*MSP-1 (ATCC, Manassas, VA, USA); mouse anti-*Pf*RNF1.1 [20]; mouse anti-*Pf*RNF1.2 [18]. The following dilutions were used: 1) IFA: rabbit anti-*Pf*s230 (1:500), rabbit anti-*Pf*39 (1:200), mouse anti-*Pf*RNF1.1 (1:20), mouse anti-*Pf*RNF1.2 (1:20), mouse anti-GFP (1:200), rabbit anti-*Pf*MSP1 (1:100); 2) Western blotting: rabbit anti-*Pf*39 (1:10,000), mouse anti-GFP (1:500), mouse anti-*Pf*RNF1.2 (1:500).

### 4.3. Parasite Culture

The gametocyte-producing strain *P. falciparum* NF54 (termed WT NF54) was used in the experiments. The cultivation of parasites and the purification of gametocytes were performed as described previously (e.g., [20,36]). Human erythrocyte concentrate and serum were purchased from the transfusion medicine department of the University Hospital Aachen, Germany. The work with human blood was approved by the University Hospital Aachen Ethics commission (EK007/13); serum samples were pooled, and the donors remained anonymous.

### 4.4. Generation of Line PfRNF1-pffnpa-GFP-BirA

The *Pf*RNF1-*pffnpa*-GFP-BirA parasite line was generated using the vector pARL-*pffnpa*-GFP-BirA as described previously [20,24,36]. *Pf*RNF1-*pffnpa*-GFP-BirA-forward-primer (primer 1; Figure S3a) and *Pf*RNF1-*pffnpa*-GFP-BirA-reverse-primer were used for gene amplification (for primer sequences, see Table S4). The presence of the vector in the transfectant line was confirmed by diagnostic PCR (Figure S3b) using the above forward primer (primer 1), as well as pARL-GFP-BirA-reverse-primer (primer 2; Figure S3a; for primer sequences, see Table S4). The amplification of the fructose bisphosphate aldolase-encoding gene *pffbpa* was used as loading control, as described previously [19].

### 4.5. Semi-Quantitative RT-PCR

To determine the transcript expression of *Pf*RNF1, total RNA was isolated from rings, trophozoites, schizonts, and immature and mature gametocytes as well as gametocytes at 30 min post-activation, and semi-quantitative RT-PCR was performed as described previously [19] using *pfrnf1*-specific primers for transcript amplification (for primer sequences, see Table S4). Stage purity was verified by the amplification of the asexual blood-stage transcript *pfama1* (apical membrane antigen 1) and the gametocyte-specific transcript *pfccp2* (LCCL domain-containing protein 2); the amplification of the *pffbpa* transcript (fructose bisphosphate aldolase) served as positive and loading control. Potential gDNA contamination was excluded by *pffbpa* amplification using RNA samples lacking reverse transcriptase (for primer sequences, see Table S4).

### 4.6. Western Blotting

Parasite lysates of lines *Pf*RNF1-*pffnpa*-GFP-BirA and WT NF54 were prepared and subjected to Western blotting as described previously [20,24,36]. *Pf*RNF1 was detected by immunoblotting with mouse anti-*Pf*RNF1.2 antibody, and *Pf*RNF1-GFP-BirA was detected using mouse anti-GFP antibody. Immunoblotting with antibodies against the endoplasmic reticulum-resident protein *Pf*39 served as a loading control. Lysates of non-infected red blood cells or WT NF54 served as negative controls. For the detection of primary antibodies, goat anti-mouse and anti-rabbit alkaline phosphatase-conjugated secondary antibodies (1:5000; Sigma-Aldrich, Taufkirchen, Germany) were used. Biotinylated proteins were labeled using alkaline phosphatase-conjugated streptavidin (1:1000; Sigma-Aldrich, Taufkirchen, Germany).

### 4.7. Indirect Immunofluorescence Assay

Methanol-fixed monolayers of blood-stage parasites of lines *Pf*RNF1-*pffnpa*-GFP-BirA and WT NF54 were subjected to IFA as described previously [20,36]. *Pf*RNF1 was detected by immunolabeling with anti-*Pf*RNF1.1 and anti-*Pf*RNF1-2 antibodies, and *Pf*RNF1-GFP-BirA was detected using mouse anti-GFP antibody. Asexual blood stages and gametocytes were highlighted by anti-*Pf*MSP1 and anti-*Pf*s230 antisera; sera from non-immunized mice were used for negative control. For the detection of primary antibodies, goat anti-mouse Alexa Fluor 488 and anti-rabbit Alexa Fluor 594 (Invitrogen, Karlsruhe, Germany) were used. The parasite nuclei were stained with Hoechst 33342 (1:5000; Invitrogen, Karlsruhe, Germany).

### 4.8. BioID-MS Analysis

Percoll-enriched immature gametocytes of lines *Pf*RNF1-*pffnpa*-GFP-BirA and WT NF54 were treated with 50 µM biotin for 24 h. The cells were subsequently harvested, processed by single-pot solid-phase-enhanced sample preparation, and subjected to liquid chromatography-mass spectrometry analysis, followed by label-free quantification as described previously [20,24,36]. BioID-MS was performed on three independent streptavidin-purified protein samples with three technical replicates for each sample. Only peptides with a minimum length of 7 amino acids were considered. Proteins had to be identified by at least two peptides and present in all three biological replicates with at least a two-fold enrichment compared to the controls. Statistical analysis of the data was conducted using Student’s *t*-test, which was corrected by the Benjamini–Hochberg (BH) method for multiple hypothesis testing (FDR of 0.01). Proteins with a putative signal peptide were excluded from further investigations.

### 4.9. Bioinformatics

The 3D structure of *Pf*RNF1 was predicted using the AlphaFold program (https://alphafold.ebi.ac.uk; see entry O97260; accessed on 19 December 2024 [37,38]). Gene expression, protein function, and GO term analysis were performed using the database PlasmoDB (http://plasmoDB.org; accessed on 13 January 2025 [23]). Transcriptomic profiling was carried out using the Malaria Cell Atlas database (https://www.malariacellatlas.org; accessed on 13 January 2025 [39]) with UMAP settings. Network analysis was conducted using the STRING database (version 11.0; https://string-db.org; accessed on 20 December 2024 [40]) utilizing the Markov Clustering algorithm and default settings.

### 4.10. Data Availability

The mass spectrometry proteomics data were deposited in the ProteomeXchange Consortium (http://proteomecentral.proteomexchange.org; accessed on 2 June 2025) via the jPOST partner repository with the dataset identifiers PXD040384 for ProteomeXchange and JPST002050 for jPOST.

## Data Availability

The mass spectrometry proteomics data are available in the ProteomeXchange Consortium (http://proteomecentral.proteomexchange.org; accessed on 2 June 2025) via the jPOST partner repository at PXD040384 (ProteomeXchange) and JPST002050 (jPOST).

## Supplementary Materials

The following supporting information can be downloaded at: https://www.mdpi.com/article/10.3390/ijms26125470/s1.

## Abbreviations

The following abbreviations are used in this manuscript:

aGC	Activated gametocyte
AMA1	Apical membrane antigen 1
Ap2	Apetala 2
BioID-MS	Proximity-dependent biotin identification with mass spectrometry
CCP2	LCCL domain-containing protein 2
FBPA	Fructose bisphosphate aldolase
FD	Female development
GD	Gametocyte development
GDV1	Gametocyte development protein 1
GFP	Green fluorescent protein
HP1	Heterochromatin protein 1
IFA	Indirect immunofluorescence assay
imGC	Immature gametocyte
MD	Male development
mGC	Mature gametocyte
niRBC	Non-infected red blood cell
PCR	Polymerase chain reaction
RBP	RNA-binding protein
RBUL	RNA-binding E3 ubiquitin ligase
RI	Ring stage
RNF1	RING finger protein 1
RT-PCR	Reverse transcriptase PCR
SZ	Schizont
TSA	Trichostatin A
TZ	Trophozoite
UPS	Ubiquitin proteasome system
WT NF54	Wildtype strain NF54
ZFP	Zinc finger protein
ZNF4	Zinc finger protein 4
