# Supplementary material for: The Plasmodium falciparum RING Finger Protein PfRNF1 Forms an Interaction Network with Regulators of Sexual Development
Source: Int J Mol Sci. 2025 Jun 7;26(12):5470. doi: 10.3390/ijms26125470 (PMC12193022; doi:10.3390/ijms26125470)
Supplement: Supplementary file 1 [file ijms-26-05470-s001.zip › Farrukh et al-IJMS-Figure S1.pdf]

Farrukh et al., Figure S1

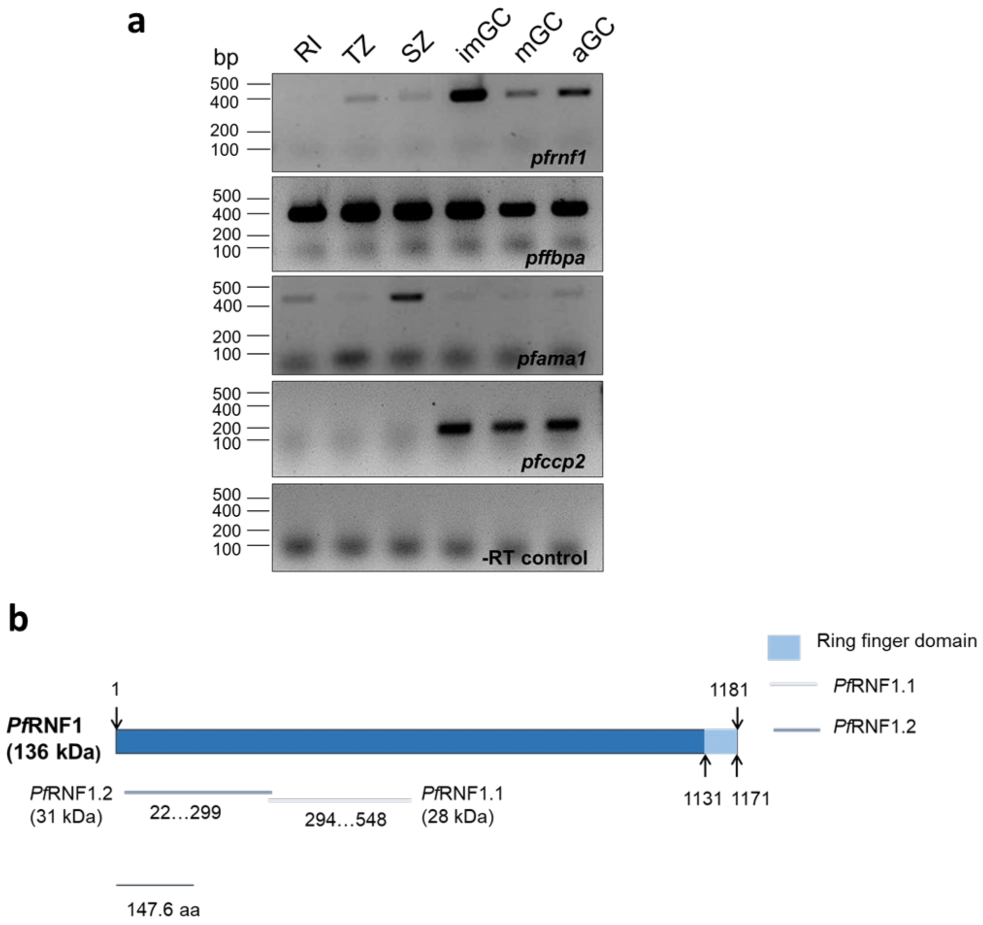

Figure S1. Transcript expression of *Pf*RNF1 in the blood stages of *P. falciparum*. **(a)** Transcript expression analysis of *Pf*RNF1 in blood stage parasites. Complementary DNA from rings (RI), trophozoites (TZ), schizonts (SZ), as well as immature (imGC), mature (mGC), and gametocytes at 30 min post-activation (aGC) of WT NF54 were subjected to diagnostic RT-PCR using *pfrnf1*-specific primers. Transcript amplification of *pffbpa* was used as housekeeping control, transcript amplification of *pfama1* and *pfccp2* served to control the specificity of the asexual blood stage and gametocyte samples, respectively. Samples without reverse transcriptase (-RT) served as genomic DNA controls. **(b)** Schematic depicting *Pf*RNF1. The RING finger domain (light blue box) and the regions homologous to the recombinant peptides *Pf*RNF1.1 (28 kDa) and *Pf*RNF1.2 (31 kDa) are highlighted.
