# Supplementary material for: The Plasmodium falciparum RING Finger Protein PfRNF1 Forms an Interaction Network with Regulators of Sexual Development
Source: Int J Mol Sci. 2025 Jun 7;26(12):5470. doi: 10.3390/ijms26125470 (PMC12193022; doi:10.3390/ijms26125470)
Supplement: Supplementary file 1 [file ijms-26-05470-s001.zip › Farrukh et al-IJMS-Figure S2.pdf]

Farrukh et al., Figure S2

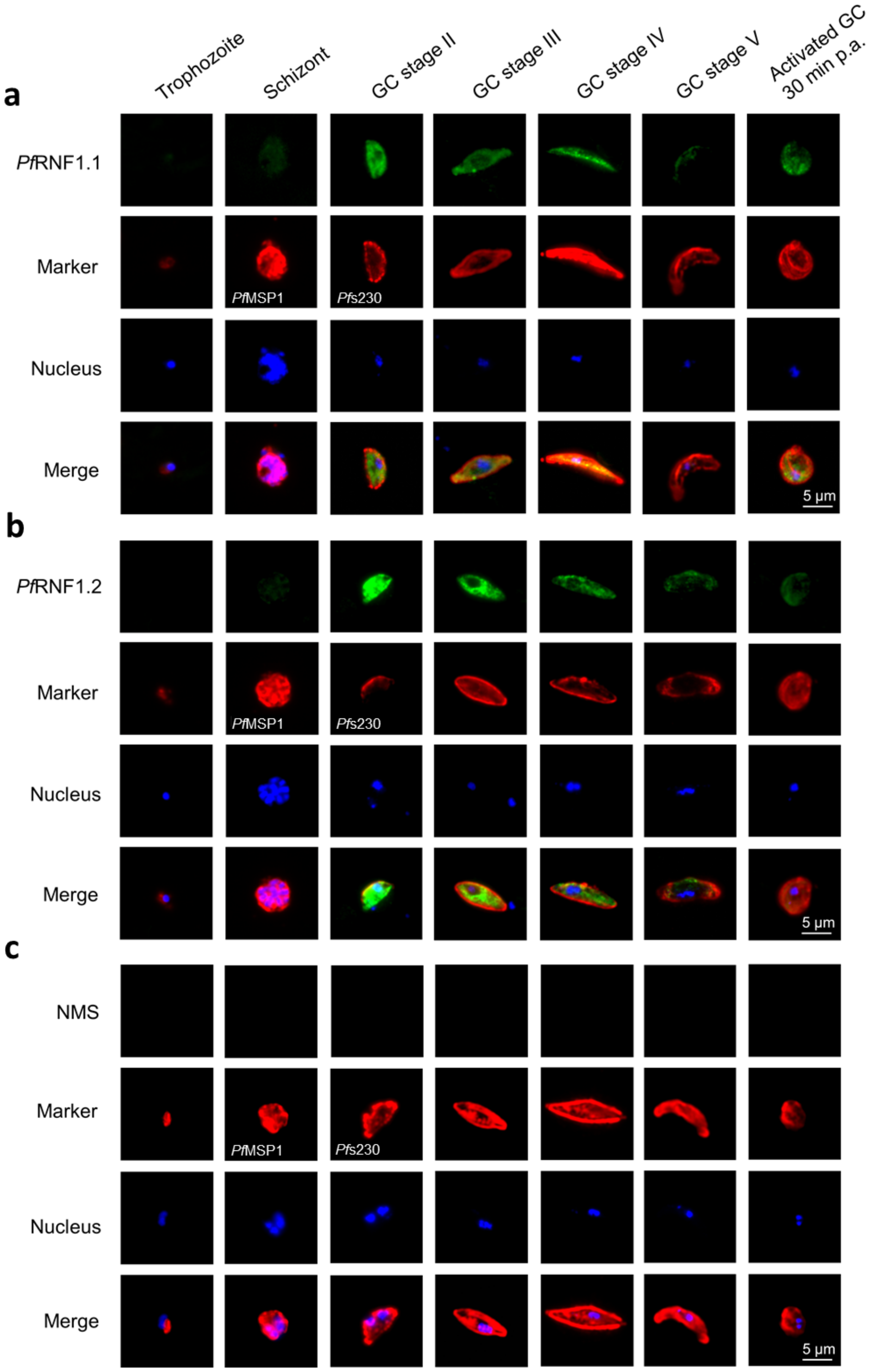

Figure S2. Protein expression and subcellular localization of *Pf*RNF1. Methanol-fixed rings (RI), trophozoites (TZ), schizonts (SZ), and immature (imGC), mature (mGC), and gametocytes at 30 min post-activation (aGC) of WT NF54 were immunolabelled with either mouse anti-*Pf*RNF1.1 **(a)** or mouse anti-*Pf*RNF1.2 antisera **(b)** (green). NMS served as a negative control **(c)**. Asexual blood stages and gametocytes were highlighted with rabbit antisera directed against *Pf*MSP1 and *Pf*s230, respectively (red); nuclei were highlighted with Hoechst 33342 nuclear stain (blue). Bar, 5 µm.
