# Supplementary material for: The Plasmodium falciparum RING Finger Protein PfRNF1 Forms an Interaction Network with Regulators of Sexual Development
Source: Int J Mol Sci. 2025 Jun 7;26(12):5470. doi: 10.3390/ijms26125470 (PMC12193022; doi:10.3390/ijms26125470)
Supplement: Supplementary file 1 [file ijms-26-05470-s001.zip › Farrukh et al-IJMS-Figure S3.pdf]

Farrukh et al., Figure S3

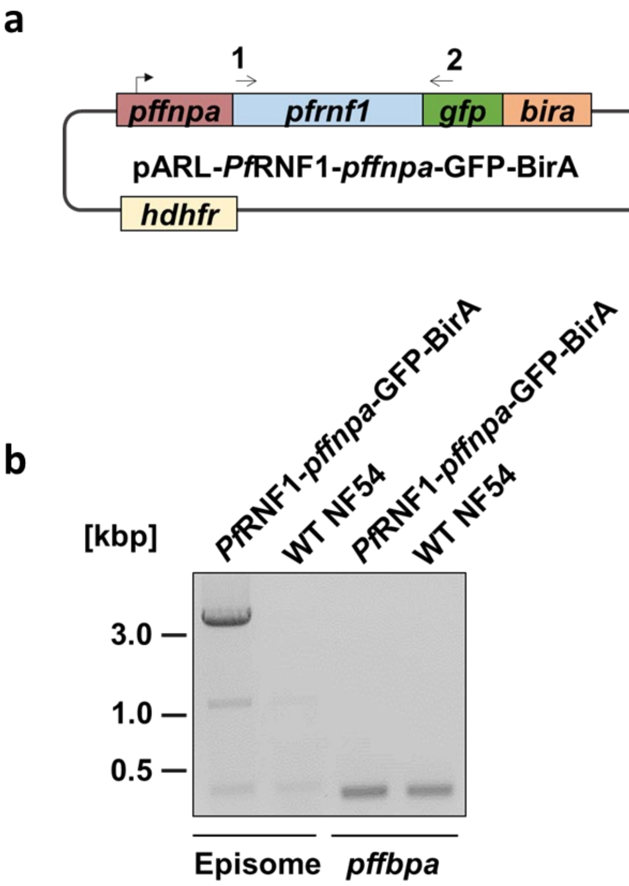

Figure S3. Generation of line *Pf*RNF1-*pffnpa*-GFP-BirA. **(a)** Schematic depicting vector pARL-*Pf*RNF1-*pffnpa*-GFP-BirA, comprising the *pffnpa* promoter, respectively. BirA, *E. coli* biotin ligase; GFP, green fluorescent protein; *hdhfr*, human dihydrofolate reductase encoding gene referring resistance to WR99210. **(b)** Verification of vector uptake in line *Pf*RNF1-*pffnpa*-GFP-BirA. The presence of vector pARL-*Pf*RNF1-*pffnpa*-GFP-BirA (primers 1 and 2; 3,775 bp) in line *Pf*RNF1-*pffnpa*-GFP-BirA was detected by diagnostic PCR. WT NF54 served as negative control; amplification of *pffbpa* (378 bp) served as loading control.
