# Supplementary material for: The Plasmodium falciparum RING Finger Protein PfRNF1 Forms an Interaction Network with Regulators of Sexual Development
Source: Int J Mol Sci. 2025 Jun 7;26(12):5470. doi: 10.3390/ijms26125470 (PMC12193022; doi:10.3390/ijms26125470)
Supplement: Supplementary file 1 [file ijms-26-05470-s001.zip › Farrukh et al-IJMS-Figure S4.pdf]

Farrukh et al., Figure S4

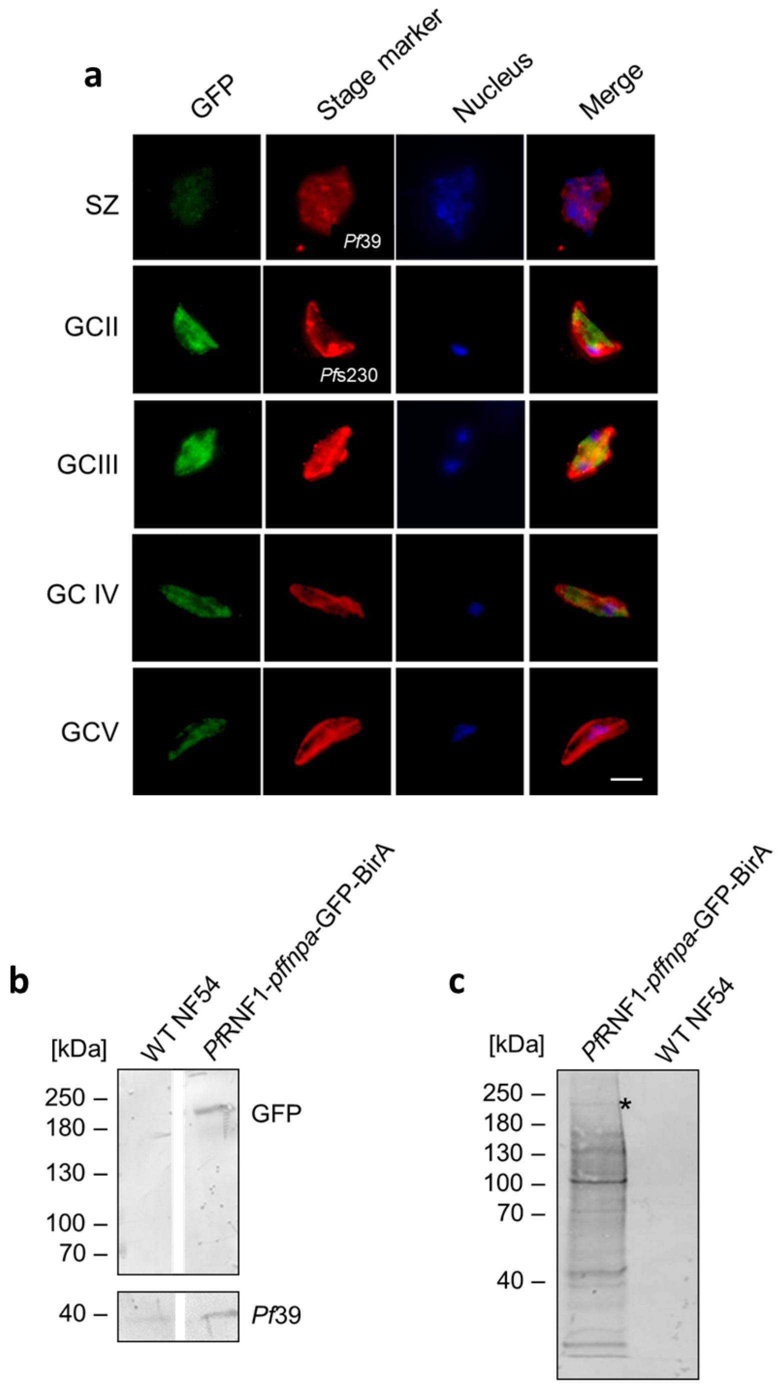

Figure S4. Verification of line *Pf*RNF1-*pffnpa*-GFP-BirA. **(a)** Subcellular localization of *Pf*RNF1-GFP-BirA in gametocytes. Methanol-fixed schizonts (SZ) and gametocyte (GC) stages II–V of line *Pf*RNF1-*pffnpa*-GFP-BirA were immunolabelled with mouse anti-GFP antibodies (green) to detect *Pf*RNF1-GFP-BirA. Gametocytes were highlighted with rabbit anti-*Pf*s230 antisera (red); nuclei were highlighted with Hoechst 33342 nuclear stain (blue). Bar, 5 µm. **(b)** *Pf*RNF1-GFP-BirA expression in blood stage parasites. Gametocyte lysate from line *Pf*RNF1-*pffnpa*-GFP-BirA was immunoblotted with mouse anti-GFP antibody to detect *Pf*RNF1-GFP-BirA (~200 kDa). WT NF54 lysate served as negative control and immunoblotting with rabbit antisera directed against the ER-resident *Pf*39 (~39 kDa) served as loading control. **(c)** Protein biotinylation in line *Pf*RNF1-*pffnpa*-GFP-BirA. Immature gametocytes of line *Pf*RNF1-*pffnpa*-GFP-BirA were incubated with 50 µM biotin for 24 h and lysates were immunoblotted using alkaline phosphatase-conjugated streptavidin to detect biotinylated proteins. The asterisk marks biotinylated *Pf*RNF1-GFP-BirA (~200 kDa). Biotin-treated WT NF54 served as negative control.
