# Supplementary material for: The Plasmodium falciparum RING Finger Protein PfRNF1 Forms an Interaction Network with Regulators of Sexual Development
Source: Int J Mol Sci. 2025 Jun 7;26(12):5470. doi: 10.3390/ijms26125470 (PMC12193022; doi:10.3390/ijms26125470)
Supplement: Supplementary file 1 [file ijms-26-05470-s001.zip › Farrukh et al-IJMS-Table S4-primers.pdf]

Table S4: List of primers used in this study

Line *Pf*RNF1-pffnpa-GFP-BirA - Insert amplification	
*Pf*RNF1-*pffnpa*-GFP-BirA-forward-primer	atgcatggtaccATGGCTCATAAAGTAAAAAAAGAAAAAAAAAC
*Pf*RNF1-*pffnpa*-GFP-BirA-reverse-primer	atgcatcctaggCTTCTTATAACTATTTCGAAGAT

Line *Pf*RNF1-pffnpa-GFP-BirA – confirmation of integration	
*Pf*RNF1-pffnpa-GFP-BirA-forward-primer	atgcatggtaccATGGCTCATAAAGTAAAAAAAGAAAAAAAAAC
pARL-GFP-BirA-reverse-primer	CAAGTGTTGGCCATGGAA

Diagnostic RT-PCR	
*Pf*RNF1 – Forward-primer	CAAACGCATGCAAAAGAAGA
*Pf*RNF1 – Reverse-primer	GTATGAGTGCCCTCCGAAAA
*Pf*AMA1 – Forward primer	CGGTAGCTACGGGAAATCAA
*Pf*AMA1 – Reverse primer	AGGGCAAACTTTTTCCCAGT
*Pf*CCP2 – Forward primer	AGTTGTTGATGGGCTTTTGG
*Pf*CCP2 – Reverse primer	ATTCGGTGCCATTAGGGTTA
*Pf*FBPA – Forward primer	TAGATGGATTAGCAGAAAGATGC
*Pf*FBPA – Reverse primer	AGAAACCAACATCTTGAGTAGTGG
